# Design of a scandium-integrated MOF hybrid hydrogel for simultaneous dye adsorption and antibacterial activity

**DOI:** 10.3389/fchem.2025.1659983

**Published:** 2025-08-22

**Authors:** Abdullah A. Aseeri, Fadhil Faez Sead, Farag M. A. Altalbawy, Nawfal Yousif, Ahmed Salih Sahib, Zahraa Saad Abdulali, Mariem Alwan, Mahmood Jawad, Hiba Mushtaq, Aseel Smerat

**Affiliations:** ^1^ Department of Clinical Laboratory Sciences, College of Applied Medical Sciences, King Khalid University, Abha, Saudi Arabia; ^2^ Department of Dentistry, College of Dentistry, The Islamic University, Najaf, Iraq; ^3^ Department of Chemistry, University College of Duba, University of Tabuk, Tabuk, Saudi Arabia; ^4^ Department of Radiology Techniques, Health and Medical Techniques College, Alnoor University, Nineveh, Iraq; ^5^ College of pharmacy, Ahl Al Bayt University, Karbala, Iraq; ^6^ Department of Pharmacology and Toxicology, College of Pharmacy, University of Kerbala, Karbala, Iraq; ^7^ College of Health and Medical Technology, National University of Science and Technology, Dhi Qar, Iraq; ^8^ Pharmacy College, Al-Farahidi University, Baghdad, Iraq; ^9^ Department of Pharmacy, Al-Zahrawi University College, Karbala, Iraq; ^10^ Gilgamesh Ahliya University, Baghdad, Iraq; ^11^ Faculty of Educational Sciences, Al-Ahliyya Amman University, Amman, Jordan; ^12^ Department of Biosciences, Saveetha School of Engineering, Saveetha Institute of Medical and Technical Sciences, Chennai, India

**Keywords:** scandium MOF, hydrogel composite, wastewater treatment, phenol red adsorption, Congo red removal, antimicrobial hydrogel, oxidized pectin, chitosan

## Abstract

In this study, a novel hybrid hydrogel incorporating a scandium-based metal-organic framework (scandium-integrated MOF-hydrogel hybrid) was developed using scandium nitrate, 1,4-naphthalenedicarboxylic acid, oxidized pectin, and chitosan. The synthesized scandium-integrated MOF-hydrogel hybrid demonstrated remarkable dual-functionality in both the adsorption of hazardous dye pollutants and the inhibition of pathogenic bacteria commonly found in wastewater. Characterization of the scandium-integrated MOF-hydrogel hybrid was performed using FT-IR, XRD, SEM, EDAX, CHNO elemental, BET, and XPS analyses, confirming successful MOF integration and a porous, reactive surface. Adsorption experiments showed significant uptake capacities for phenol red and Congo red dyes, reaching up to 40 mg and 60 mg under different physicochemical conditions, including variations in pH, temperature, adsorbent dose, and contact time. Antibacterial assessments against four wastewater-derived bacterial strains revealed minimum inhibitory concentrations ranging from 2 to 64 μg/mL and minimum bactericidal concentrations from 4 to 128 μg/mL. These results highlight the hydrogel’s potential as a multifunctional material for simultaneous pollutant removal and microbial decontamination in wastewater treatment systems.

## 1 Introduction

The escalating environmental impact of industrial and agricultural activities has led to the release of a wide range of pollutants into natural water bodies, posing severe threats to both ecosystems and public health (Saxena, 2025). Among these contaminants, synthetic dyes and pathogenic microorganisms are of particular concern (Tkaczyk et al., 2020). Dyes such as phenol red and Congo red are widely used in textile, pharmaceutical, food, and cosmetic industries due to their intense color and chemical stability (Islam et al., 2018; Siddiqui et al., 2023). However, their persistence in aquatic environments, resistance to degradation, and toxicological effects on living organisms make them particularly hazardous (Ismail et al., 2019). Even at low concentrations, these dyes can interfere with photosynthetic processes in aquatic life and may also exhibit mutagenic and carcinogenic properties in humans (Ramamurthy et al., 2024).

In addition to chemical pollutants, untreated or poorly treated wastewater often contains a variety of pathogenic bacteria, including drug-resistant strains (Werkneh and Islam, 2023). These microorganisms can spread waterborne diseases, contaminate agricultural land, and undermine the effectiveness of water treatment infrastructures (Izah and Ogwu, 2025). Traditional wastewater treatment technologies often fall short in effectively removing both types of pollutants simultaneously, necessitating the development of innovative, multifunctional materials that can address these challenges with high efficiency and environmental compatibility (Khan et al., 2023).

In recent years, metal-organic frameworks (MOFs) have gained significant attention in the field of water purification due to their high surface area, tunable pore structures, and flexible chemical functionality (Song et al., 2023). These crystalline porous materials, composed of metal nodes and organic linkers, offer promising adsorption and catalytic properties for the removal of a broad spectrum of pollutants (Mane et al., 2024). However, the practical application of MOFs in aqueous media is limited by their poor water stability, high production cost, and challenges in separation and recovery (An et al., 2024).

To overcome these limitations, researchers have explored the incorporation of MOFs into biopolymeric hydrogel matrices (Yao et al., 2024). Hydrogels, with their hydrophilic, porous, and mechanically stable networks, provide an ideal scaffold to host MOFs while improving their dispersibility, biocompatibility, and handling (Sharifabad et al., 2025). Moreover, the combination of natural polymers such as chitosan and oxidized pectin not only enhances the structural stability of the composite material but also contributes additional adsorption and antimicrobial functionalities due to the presence of reactive functional groups (e.g., hydroxyl, carboxyl, and aldehyde groups) (Guo et al., 2024; Zhang W. et al., 2023).

Scandium-based MOFs, though relatively underexplored, offer unique chemical characteristics due to the trivalent nature and moderate ionic radius of Sc^3+^, enabling the formation of highly coordinated, robust frameworks (Barsukova et al., 2018). Additionally, scandium ions have demonstrated antibacterial properties, suggesting potential synergistic effects when combined with biopolymers in hybrid structures (Shaji et al., 2024; Titova et al., 2025).

In this work, we report the fabrication of a novel hydrogel composite containing a scandium-MOF network synthesized from scandium nitrate and 1,4-naphthalenedicarboxylic acid (NDC) as the organic linker, and supported by a biopolymeric matrix of chitosan and oxidized pectin (scandium-integrated MOF-hydrogel hybrid). The resulting material integrates the adsorptive capacity of the MOF with the functional and antimicrobial characteristics of the hydrogel matrix. The scandium-integrated MOF-hydrogel hybrid was thoroughly characterized using FTIR, SEM, XRD, BET, XPS, and EDX mapping, and its performance was evaluated through adsorption experiments for phenol red and Congo red under various operational conditions, as well as through antimicrobial assays (MIC and MBC) against common wastewater-derived pathogenic strains.

Although various MOFs have been incorporated into polymeric hydrogels for environmental applications, the integration of scandium-based MOFs into biopolymer matrices remains largely unexplored, especially in the context of dual-functionality for simultaneous dye adsorption and antibacterial activity. Only a few studies have considered Sc-MOF composites in water purification, and most lack a biodegradable or biocompatible hydrogel support (e.g., chitosan or oxidized pectin). Furthermore, existing works often focus either on adsorption or antimicrobial function individually, not both.

The novelty of this study lies in (i) the use of scandium nitrate and 1,4-naphthalenedicarboxylic acid to form a stable, crystalline Sc-MOF under microwave-assisted conditions, (ii) its integration into a natural biopolymer hydrogel comprising oxidized pectin and chitosan, and (iii) the demonstration of simultaneous dye removal and antibacterial efficacy in a single multifunctional platform. To the best of our knowledge, this is the first report of a Sc-MOF hydrogel exhibiting this combination of properties, thereby addressing a key gap in environmentally friendly materials for advanced wastewater treatment.

## 2 Materials and methods

### 2.1 Materials and instrumentation

All reagents were of analytical grade and used without further purification. Scandium (III) nitrate, 1,4-naphthalenedicarboxylic acid (NDC, ≥98%), glacial acetic acid (≥99%), and sodium hydroxide (NaOH) were purchased from Sigma-Aldrich (United States). Chitosan (medium molecular weight, deacetylation degree >75%) was obtained from Acros Organics (Belgium). Oxidized pectin was prepared from commercial citrus pectin (Sigma-Aldrich) following a previously reported oxidation method. Congo red and phenol red dyes were obtained from Merck (Germany). All aqueous solutions were prepared using deionized water (resistivity ≥18.2 MΩ cm).

Bacterial strains including *Escherichia coli* (ATCC 25922), *Staphylococcus aureus* (ATCC 25923), *Pseudomonas aeruginosa* (ATCC 27853), and *Bacillus subtilis* (ATCC 6633) were purchased from the American Type Culture Collection (ATCC, United States).

Microwave-assisted synthesis was carried out using a CEM Discover SP microwave synthesizer (CEM Corporation, United States) operating at 300 W with real-time temperature control. Ultrasonic dispersion was performed using a BANDELIN SONOREX RK 100H ultrasonic bath (Bandelin, Germany) at a frequency of 35 kHz and power of 320 W.

For structural characterization, the following instruments were used:

Fourier-transform infrared spectroscopy (FT-IR): Spectra were recorded in the range of 4,000–400 cm^-1^ using a Bruker Alpha II FTIR spectrometer (Bruker, Germany) equipped with an ATR accessory. Each spectrum was obtained by averaging 32 scans with a spectral resolution of 4 cm^-1^ at room temperature.

Scanning electron microscopy (SEM): Surface morphology was examined using a Tescan MIRA3 field-emission SEM (Tescan, Czech Republic). Prior to SEM imaging, hydrogel samples were freeze-dried to preserve their porous structure. The dried samples were then coated with a thin layer of gold using a sputter coater (EM ACE200, Leica, Germany) to improve conductivity.

X-ray diffraction (XRD): Crystallographic analysis was performed on a PANalytical X'Pert PRO diffractometer (Malvern Panalytical, Netherlands) using Cu Kα radiation (λ = 1.5406 Å) over a 2θ range of 5°–30°.

Brunauer–Emmett–Teller (BET) surface area analysis: N_2_ adsorption–desorption isotherms were measured using a Micromeritics ASAP 2020 analyzer (Micromeritics, United States) at 77 K. For BET surface area analysis, samples were degassed under vacuum at 120 °C for 12 h to remove any adsorbed moisture or volatile compounds prior to nitrogen adsorption measurements.

X-ray photoelectron spectroscopy (XPS): Elemental composition and oxidation states were evaluated using a Thermo Scientific K-Alpha XPS system (Thermo Fisher Scientific, United States) with Al Kα radiation (hν = 1486.6 eV).

UV-Vis spectrophotometry: Dye adsorption studies and preparation of the bacterial suspension were performed using a Shimadzu UV-1800 spectrophotometer (Shimadzu, Japan) at wavelengths of 430 nm (phenol red), 497 nm (Congo red), and 625 nm (bacterial suspension).

### 2.2 Synthesis of scandium-integrated MOF-hydrogel hybrid

To synthesize the Sc-based MOF, 1 mmol of scandium nitrate and 1 mmol of 1,4-naphthalenedicarboxylic acid (NDC) were dissolved in 100 mL of a 1:1 (v/v) ethanol–water mixture under continuous stirring at 50 °C for 20 min. Subsequently, 0.1 mmol of oxidized pectin was added to the mixture, and the resulting solution was subjected to microwave irradiation at 320 W for 20 min. After the reaction, the precipitated product was separated using nanofiltration, washed thoroughly with a 1:1 ethanol:water mixture to remove unreacted precursors and residual solvent, and dried under vacuum at room temperature for 24 h. The dried powder was used for further composite hydrogel formation (Al-Khafaji et al., 2023).

To prepare the hydrogel, 1 g of medium molecular weight chitosan (with >75% degree of deacetylation) was dispersed in 100 mL of 1% (v/v) acetic acid solution under magnetic stirring for 6 h at 40 °C until a homogeneous viscous solution was obtained. Then, 250 mg of the previously synthesized Sc-NDC-oxidized pectin MOF was added to the chitosan solution. The mixture was sonicated in an ultrasonic bath for 15 min to ensure uniform dispersion of the MOF particles and then stirred magnetically for an additional 30 min (Namli et al., 2023).

The pH of the resulting solution was gradually adjusted to approximately 7.2 by dropwise addition of 1 N NaOH, promoting hydrogel formation through physical gelation. The gelled material was transferred into molds and allowed to stabilize at room temperature for 12 h. For enhanced structural stability, an optional crosslinking step was performed by adding 75 μL of 25% glutaraldehyde under mild stirring. Finally, the hydrogel was washed repeatedly with deionized water to remove any residual reagents and stored at 4 °C until further use (Al-Khafaji et al., 2023; Trombino et al., 2023).

### 2.3 Phenol red adsorption

The adsorption performance of phenol red dye was systematically investigated by varying several parameters, including the initial dye concentration, adsorbent dosage, solution pH (ranging from 4 to 10), temperature (25 °C–60 °C), and contact time (25–200 min). For each adsorption experiment, a predetermined amount of the synthesized scandium-integrated MOF-hydrogel hybrid was introduced into 25 mL of phenol red aqueous solution. The mixtures were continuously stirred at 140 rpm to ensure uniform dispersion and interaction between the adsorbent and dye molecules. Upon completion of the specified contact time, the suspensions were subjected to centrifugation at 6,000 rpm for 10 min to separate the solid adsorbent from the solution phase.

The residual concentration of phenol red in the supernatant was quantified by measuring its absorbance at 430 nm using a UV-Visible spectrophotometer. The adsorption efficiency (Re, %) was calculated according to the following equation (Mohammed Yaseen et al., 2025):

$$Re\;\left(\%\right)=\left(\frac{C0-Ce}{C0}\right)\times 100$$ (1)

C_0_ = Initial dye concentration (mg/L).

C_e_ = Equilibrium dye concentration (mg/L).

Equation 1. Calculation of absorption percentage.

All experiments were performed in triplicate to ensure reproducibility, and the average values were reported.

Centrifugation was performed to ensure complete separation of the hydrogel from the solution phase, as minor detachment of surface-bound particles or loosely held hydrogel fragments may occur during stirring. This step was necessary to avoid interference in UV-Vis measurements and ensure accurate determination of dye concentration in the supernatant. The core hydrogel structure remained macroscopically intact during all experiments.

### 2.4 Congo red adsorption

The adsorption experiments for Congo red dye were carried out under varying conditions including initial dye concentration, adsorbent dosage, solution pH, temperature, and contact time to thoroughly evaluate the adsorption capacity and behavior of the synthesized hydrogel. In each test, a specified amount of the scandium-integrated MOF-hydrogel hybrid was added to 25 mL of Congo red aqueous solution. The mixtures were continuously stirred at 140 rpm to promote effective interaction between the adsorbent and dye molecules. Following the predetermined contact time, the suspensions were centrifuged at 6,000 rpm for 10 min to separate the adsorbent from the solution phase. The concentration of residual Congo red in the supernatant was determined by measuring the absorbance at 497 nm using a UV-Visible spectrophotometer. The removal efficiency (Re, %) was calculated using the same equation applied for phenol red adsorption, where initial and equilibrium dye concentrations were used. All experiments were conducted in triplicate and averaged to ensure accuracy and reproducibility (Mohammed Yaseen et al., 2025).

### 2.5 Antimicrobial activity assay

The antimicrobial efficacy of the synthesized scandium-integrated MOF-hydrogel hybrid was evaluated against a panel of clinically relevant bacterial strains commonly found in wastewater. The bacterial strains tested included *Listeria monocytogenes, Staphylococcus aureus*, *Streptococcus agalactiae, Escherichia coli, Pseudomonas aeruginosa, and Klebsiella pneumonia*. Prior to testing, bacterial cultures were grown overnight in nutrient broth at 37 °C to reach the logarithmic growth phase.

The minimum inhibitory concentration (MIC) and minimum bactericidal concentration (MBC) of the hydrogel were determined using the broth micro dilution method. Serial dilutions of the hydrogel were prepared in sterile nutrient broth, and each well was inoculated with approximately 1 × 10^6^ CFU/mL of the bacterial suspension. The microplates were incubated at 37 °C for 24 h. MIC was defined as the lowest concentration of the scandium-integrated MOF-hydrogel hybrid that completely inhibited visible bacterial growth, as observed by the absence of turbidity. To determine the MBC, aliquots from wells showing no visible growth were plated onto nutrient agar and incubated for an additional 24 h at 37 °C. The MBC was identified as the lowest concentration of the hydrogel that resulted in no bacterial colony formation on the agar plates (Moghaddam-manesh et al., 2023; Mohammed Yaseen et al., 2025).

All experiments were performed in triplicate to ensure reproducibility, and appropriate positive and negative controls were included throughout the study.

### 2.6 Synthesis and structural characterization of Sc–MOF@Chitosan hydrogel

The successful synthesis of the scandium-based MOF and its subsequent incorporation into the chitosan-based hydrogel matrix were confirmed through a series of complementary analytical techniques. Each analysis provided specific insights into the molecular structure, surface morphology, elemental composition, crystallinity, and chemical environment of the resulting material.

**FIGURE 1:**
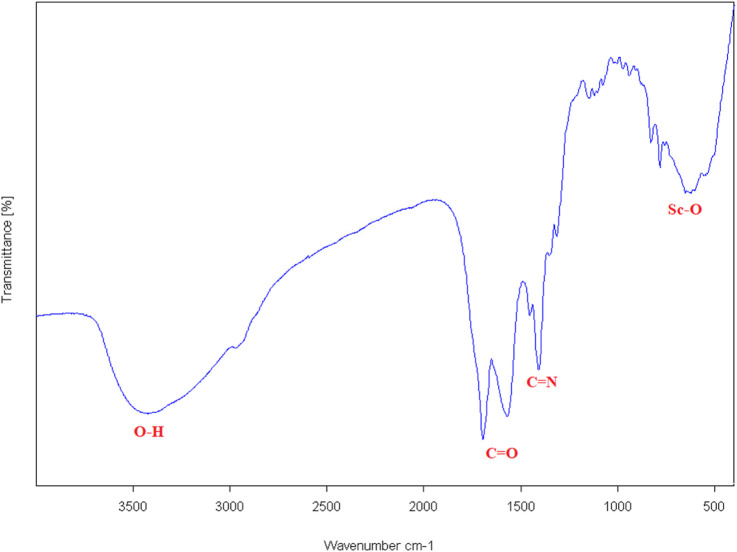
FT-IR of Scandium-integrated MOF-hydrogel hybrid.

The FT-IR spectrum of the synthesized scandium-integrated MOF-hydrogel hybrid (Figure 1) exhibited characteristic bands corresponding to the stretching vibrations of carboxylate groups (–COO^-^) at approximately 1,560–1,690 cm^-1^, confirming coordination of the NDC ligand and oxidized pectin with scandium ions. The peak observed in the 500–600 cm^-1^ region can be attributed to metal (scandium)-oxygen. After incorporation into chitosan, the spectrum of the composite, a broad band near 3,500 cm^-1^ indicated the presence of–OH groups from hydroxyl groups in chitosan, and peaks at 1,400 cm^-1^ (C=N), confirming the presence of chitosan, as indicated by the broad–OH/NH stretching band near 3,500 cm^-1^ and the characteristic C=N stretching at 1,400 cm^-1^. These features suggest possible hydrogen bonding and electrostatic interactions between the amine groups of chitosan and the carboxylate groups of the MOF components.

**FIGURE 2:**
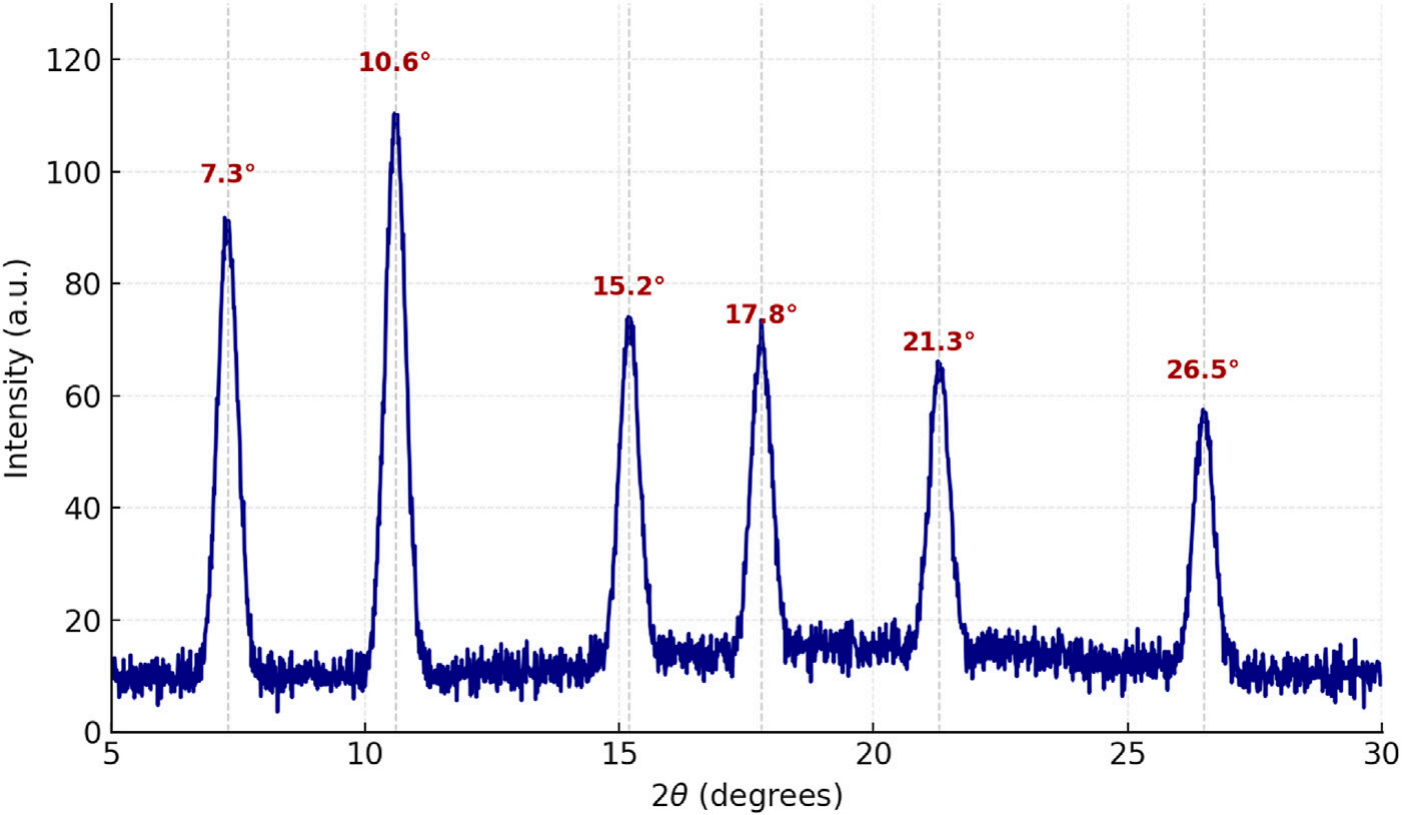
XRD of Scandium-integrated MOF-hydrogel hybrid.

The crystalline structure of the synthesized scandium-integrated MOF-hydrogel hybrid was examined using powder X-ray diffraction (XRD). The XRD pattern of the scandium-integrated MOF-hydrogel hybrid (Figure 2) exhibited distinct sharp peaks at 2θ values of approximately 7.3°, 10.6°, 15.2°, 17.8°, 21.3°, and 26.5°, indicating the formation of a highly ordered crystalline framework. These peaks correspond well to those reported for Sc-based metal–organic frameworks incorporating aromatic dicarboxylic acid ligands. Notably, the observed diffraction pattern shows strong similarity to the standard reference pattern JCPDS No. 00–056–1,475, assigned to scandium benzenedicarboxylate frameworks. The intensity and position of the major peaks confirmed the successful coordination of the scandium ion with the NDC linker, forming a three-dimensional porous network.

After incorporation into the chitosan matrix to form the hydrogel, the characteristic peaks of the MOF structure remained visible, albeit with slightly reduced intensity and some broadening. This suggests that while the MOF retained its crystalline phase, the surrounding chitosan introduced an amorphous background, which is typical for such composite hydrogel systems (Li et al., 2024).

The crystallite size of the scandium-integrated MOF-hydrogel hybrid was estimated using the Scherrer equation (Burton et al., 2009). Using the peak at 2θ ≈ 10.6°, the average crystallite size was calculated to be approximately 97 nm, indicating the formation of nanocrystalline MOF domains.

**FIGURE 3:**
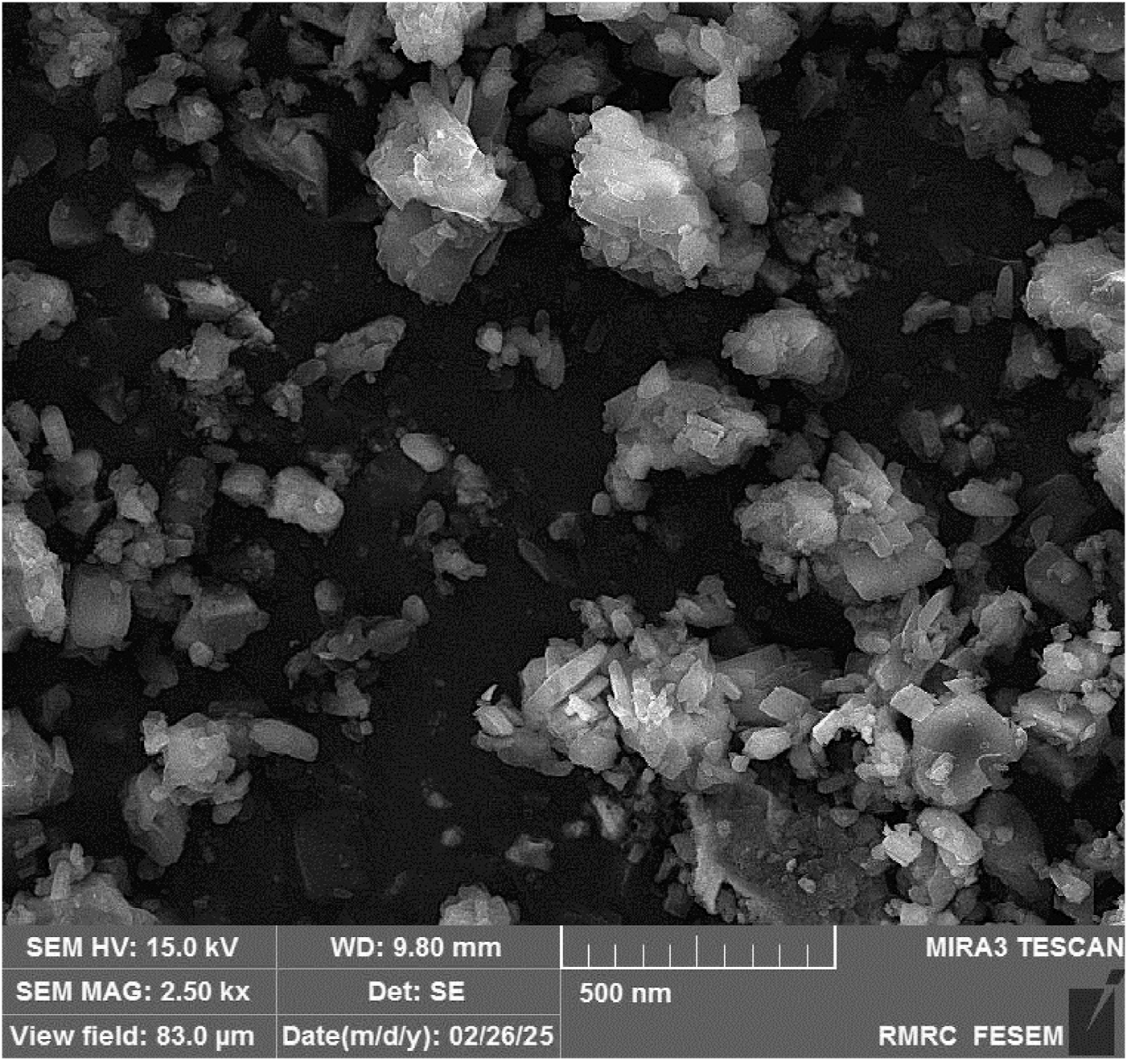
SEM of Scandium-integrated MOF-hydrogel hybrid.

SEM images of the synthesized scandium-integrated MOF-hydrogel hybrid (Figure 3) revealed a relatively uniform surface morphology. The image displays the embedded MOF particles within the porous chitosan-based hydrogel matrix. The polyhedral or rod-like nanostructures correspond to the Sc-MOF crystals, while the surrounding fibrous network is attributed to the chitosan scaffold. Although the contrast between MOF and chitosan is limited due to similar electron densities, the morphology suggests uniform dispersion.

Average MOF particle size was estimated to be approximately 93 nm by analyzing at least 50 individual particles using ImageJ software.

**FIGURE 4:**
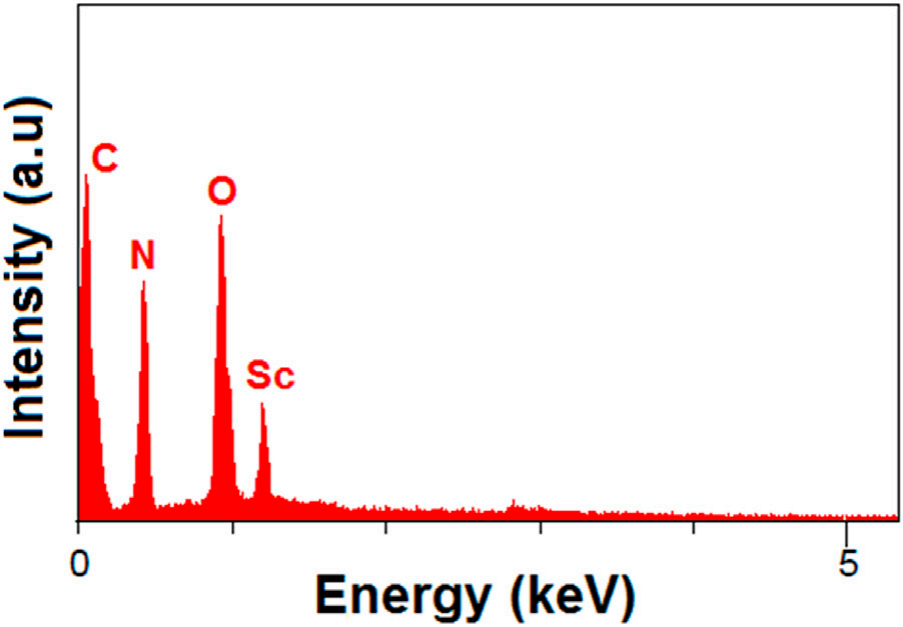
EDAX of Scandium-integrated MOF-hydrogel hybrid.

After hydrogel formation, the MOF particles were embedded within a porous, fibrous chitosan matrix. EDAX (Figure 4) and CHNO elemental analysis confirmed the distribution of scandium, oxygen, carbon, and nitrogen elements, supporting the successful incorporation of final structure. In CHNO elemental analysis, carbon was found to be 50.24%, nitrogen 5.36%, and oxygen 3.69%.

**FIGURE 5:**
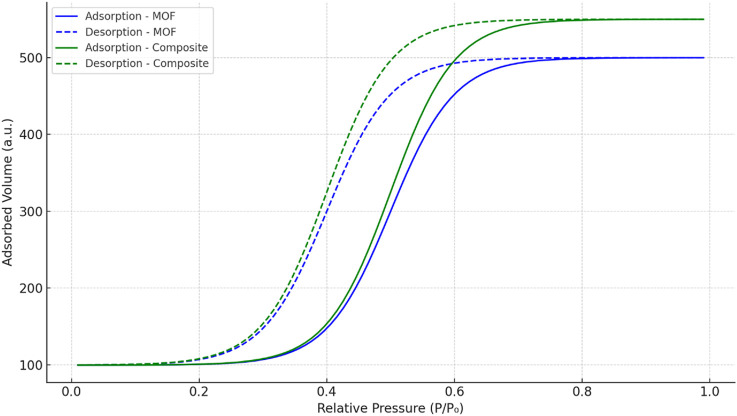
Nitrogen adsorption/desorption isotherms of MOF and Scandium-integrated MOF-hydrogel hybrid.

Nitrogen adsorption/desorption isotherms exhibited type IV characteristics with a hysteresis loop, indicating mesoporous nature (Toncón-Leal et al., 2021). The BET surface area of the pure MOF was calculated to be 1,192 m^2^/g, while the composite hydrogel showed a slightly increased value of 1,438 m^2^/g, possibly due to the formation of additional mesopores within the hydrogel network that enhance overall surface accessibility. (Figure 5).

The average pore diameter of the composite hydrogel was measured to be 1.62 nm, placing it at the lower end of the mesoporous range. This pore size is considered effective for adsorbing small-to medium-sized dye molecules, such as phenol red and Congo red, which typically exhibit molecular dimensions below 2 nm (Lu et al., 2023; Xue et al., 2023). The pore dimensions facilitate sufficient surface contact and diffusion of dye molecules into the hydrogel matrix.

**FIGURE 6:**
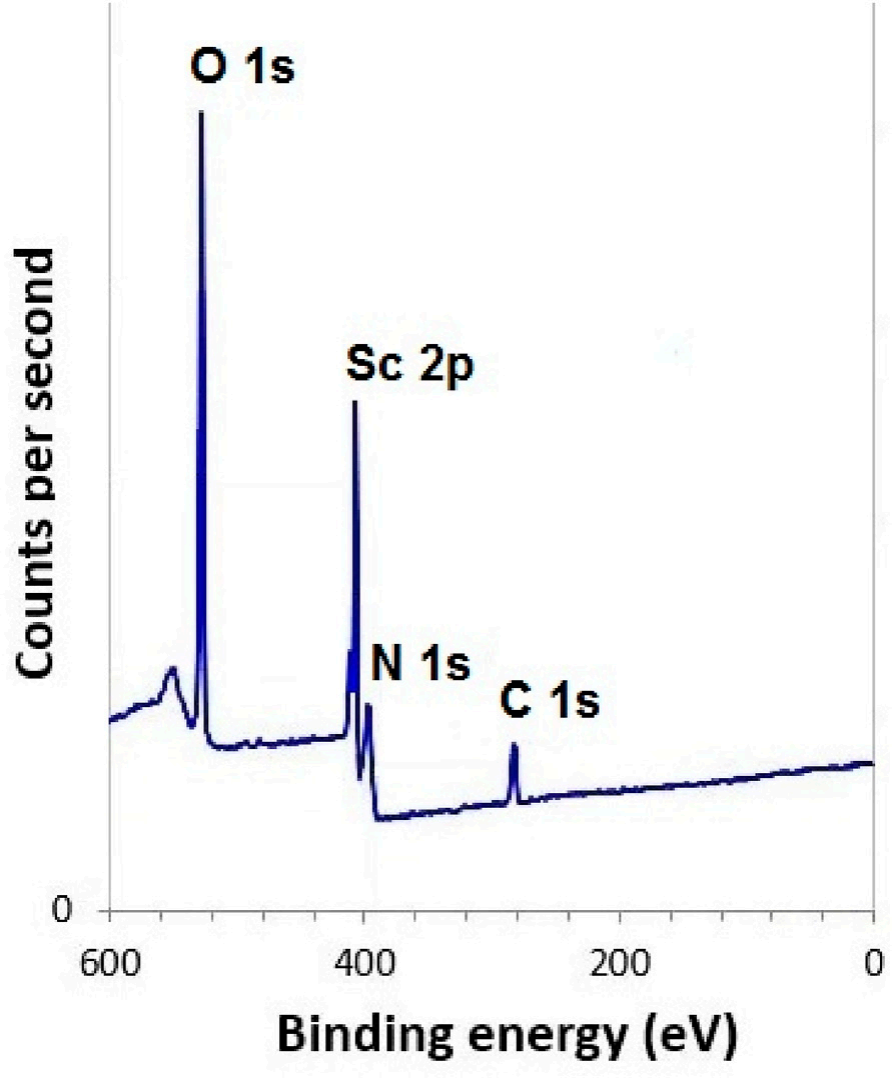
XPS of Scandium-integrated MOF-hydrogel hybrid.

The XPS survey spectrum (Figure 6) revealed the presence of Sc, C, O, and N elements. High-resolution Sc 2p peaks appeared at binding energies of approximately 401.8 eV and 407.2 eV, consistent with trivalent scandium species (Biesinger et al., 2010). The C 1s spectrum displayed distinct peaks for C–C, C-H (284.4 eV), C–O (286.6 eV), and O–C=O environments (288.3 eV) (Kumar et al., 2024), while O 1s peaks confirmed contributions from carbonyl bonds (531.5 eV), C–O–C and hydroxyl functionalities (532.1 eV) (Kwan et al., 2015). The N 1s peak (399.2 eV) supported the presence of chitosan’s nitrogen groups in the hydrogel structure (Tang et al., 2017).

**FIGURE 7:**
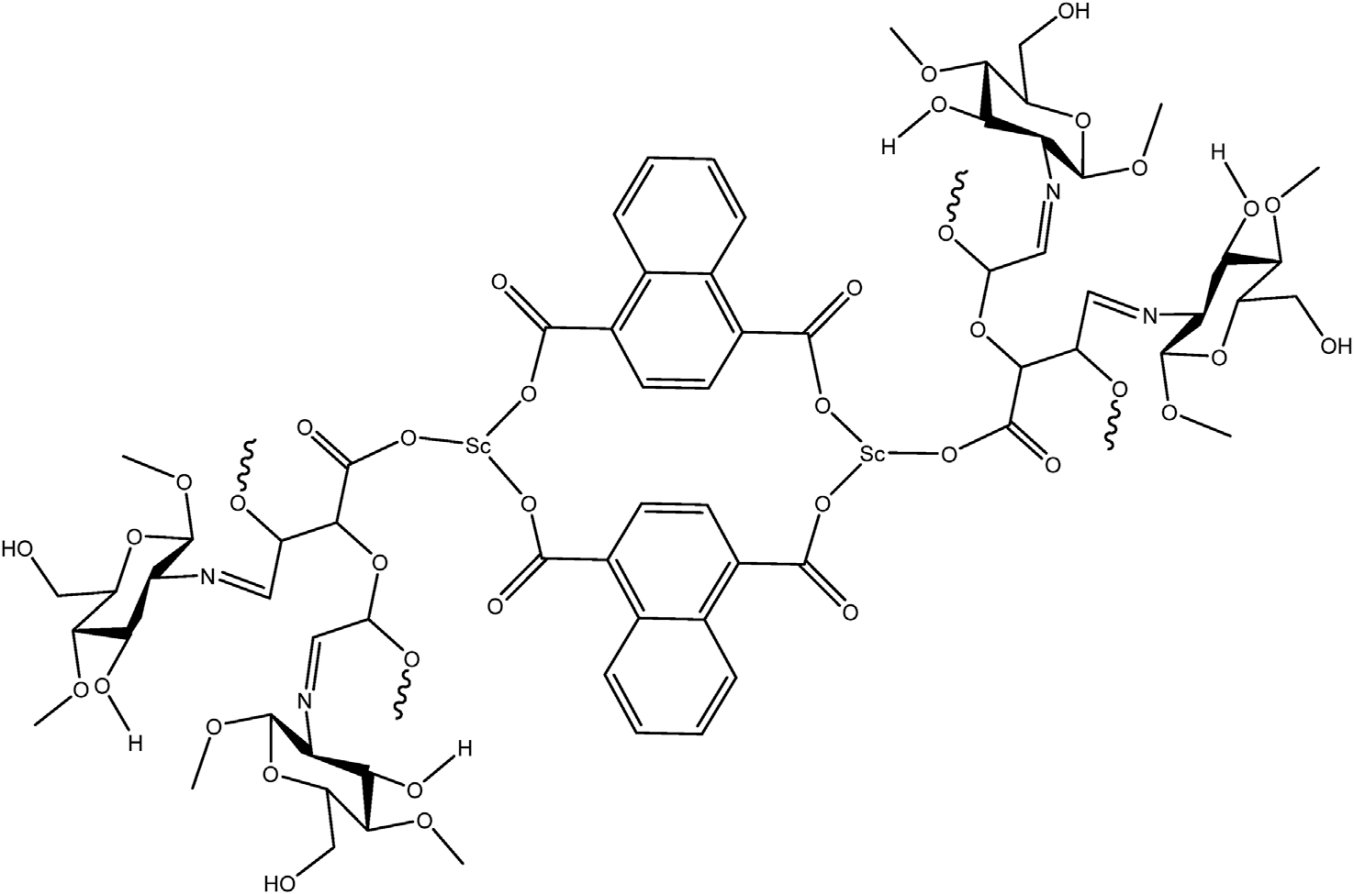
Structure of Scandium-integrated MOF-hydrogel hybrid.

Based on the experimental data and coordination behavior of the ligands, it is proposed that the scandium ion coordinates with both carboxylate groups of NDC and oxidized pectin to form a stable three-dimensional network. The oxidized pectin acts as a secondary linker, bridging adjacent Sc-centers via carboxylic and hydroxyl functionalities. In the final step, chitosan physically entraps the MOF within its polymeric chains through hydrogen bonding and electrostatic interactions, resulting in the formation of a hybrid hydrogel structure (Cavallaro et al., 2021). A schematic illustration of the proposed structure is provided in Figure 7, depicting the coordination nodes, linker arrangement, and hydrogel matrix.

Taken together, the combined results from FT-IR, XRD, SEM, EDAX, CHNO elemental, BET, and XPS analyses confirm the successful synthesis of the target Sc-based MOF structure and its stable integration within the chitosan matrix. The retention of key crystalline features, the uniform dispersion of elements, and the preservation of mesoporosity after hydrogel formation demonstrate the structural integrity and compatibility of the MOF with the biopolymeric network.

These characterization findings clearly indicate that the applied synthesis strategy was effective in producing a nanostructured hybrid material with desirable physicochemical properties (Arif et al., 2024). The resulting hydrogel presents a favorable morphology, chemical composition, and surface area that support its potential in adsorption and biological applications, validating the functional design of the composite for environmental and biomedical use (Alshangiti et al., 2023).

### 2.7 Adsorption performance

#### 2.7.1 Results of adsorption

The adsorption capability of the synthesized scandium-integrated MOF-hydrogel hybrid was systematically investigated toward two model organic dyes, namely, phenol red and Congo red, under various operational conditions. The experiments were designed to assess the effects of initial dye concentration (100–1,000 mg/L), adsorbent dose (10–100 mg), solution pH (4–10), temperature (25 °C–50 °C), and contact time (30–180 min) on dye removal efficiency.

In each experiment, a known amount of the scandium-integrated MOF-hydrogel hybrid was added to 25 mL of dye solution, and the mixture was stirred at 140 rpm. After the specified time interval, the solution was centrifuged, and the absorbance of the supernatant was measured using a UV–Vis spectrophotometer at 430 nm for phenol red and 497 nm for Congo red, respectively. The dye removal efficiency (Re, %) were calculated using standard equations (Equation 1).

The results demonstrated that the adsorption capacity increased with longer contact times, while optimum performance was observed at pH of 8 °C and 50 °C.

**FIGURE 8:**
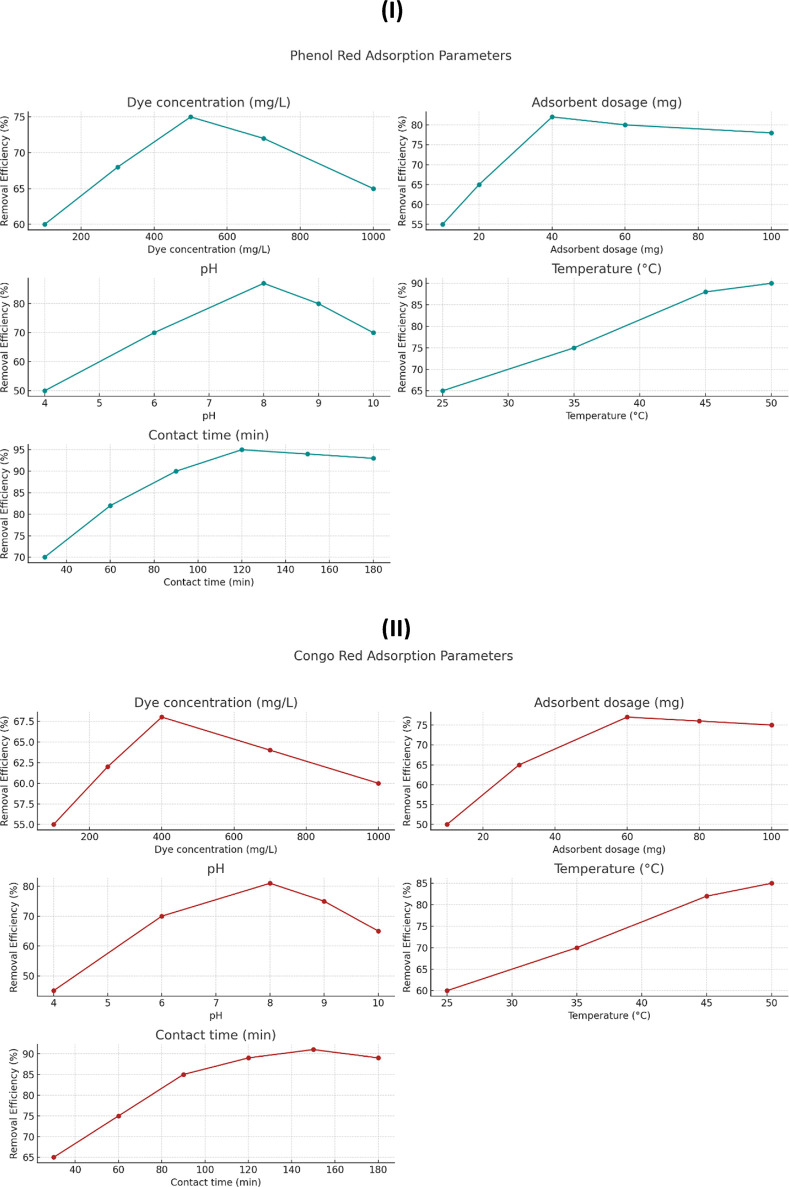
Effect of operational parameters on dye removal efficiency: **(A)** Phenol red; **(B)** Congo red. Parameters include dye concentration, adsorbent dosage, pH, temperature, and contact time.

To better visualize the effect of each variable on dye removal efficiency, the data from Tables 1, 2 were converted into plots, as shown in Figure 8. These graphs illustrate how changes in adsorbent dosage, initial dye concentration, pH, temperature, and contact time influence the adsorption efficiency for both phenol red (Figures 8A) and Congo red (Figures 8B). The trends observed from the plots support the selection of optimal parameters, as increases in temperature and contact time, for example, clearly correlate with improved dye removal. This graphical approach also reveals the saturation points beyond which further increases in adsorbent dosage or dye concentration result in marginal efficiency gains.

**TABLE 1:** Adsorption conditions and removal efficiencies for phenol red.

Parameter	Tested range	Optimal value	Removal efficiency (%)
Dye concentration (mg/L)	100–1,000	500	75
Adsorbent dosage (mg)	10–100	40	82
pH	4–10	8	87
Temperature (°C)	25–50	50	90
Contact time (min)	30–180	120	95

**TABLE 2:** Adsorption conditions and removal efficiencies for Congo red.

Parameter	Tested range	Optimal value	Removal efficiency (%)
Dye concentration (mg/L)	100–1,000	400	68
Adsorbent dosage (mg)	10–100	60	77
pH	4–10	8	81
Temperature (°C)	25–50	50	85
Contact time (min)	30–180	150	91

**FIGURE 9:**
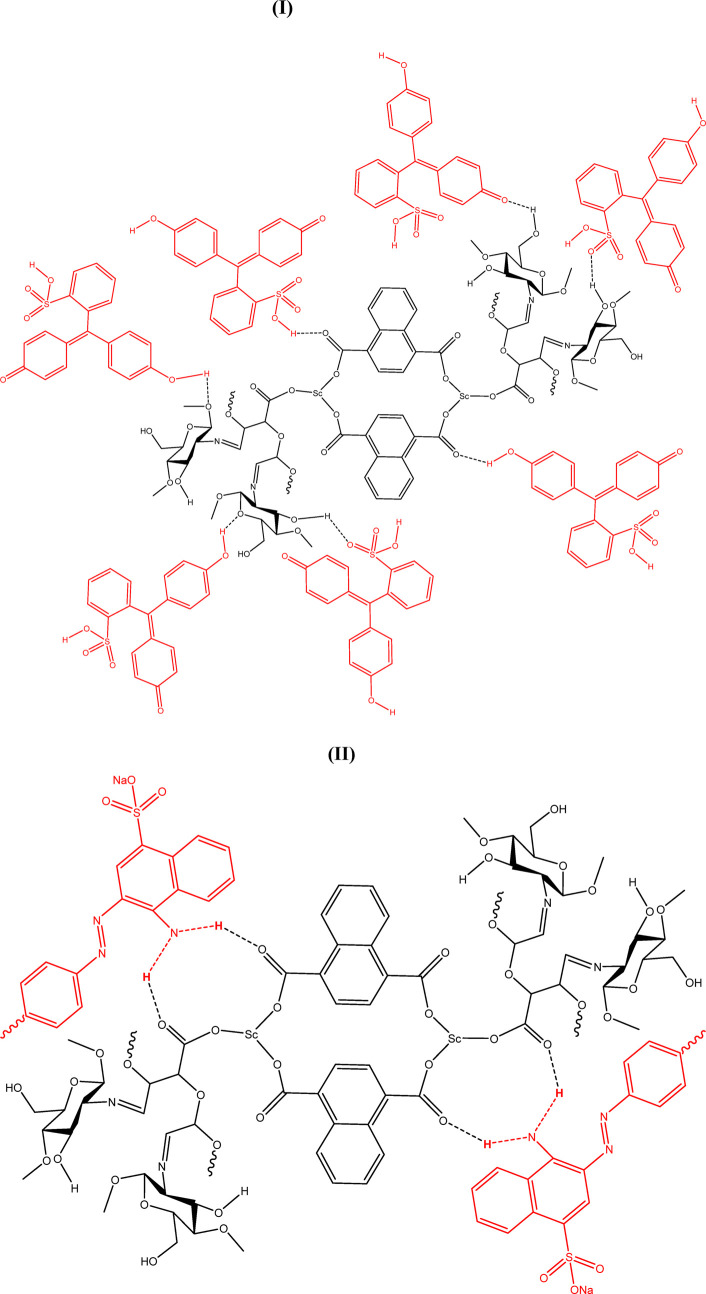
Adsorption of phenol red **(I)** and Congo red **(II)** using scandium-integrated MOF-hydrogel hybrid.

For the adsorption of phenol red (Figures 9I) and Congo red (Figures 9II) using scandium-integrated MOF-hydrogel, the following structures are proposed based on hydrogen bond formation. The following adsorption mechanisms are proposed based on the chemical functionality of the hydrogel and dyes, as well as pre-adsorption characterization data (FTIR, XPS, and BET). Since no post-adsorption spectral data were collected, these models are hypothetical and should be interpreted as illustrative representations of plausible binding interactions.

#### 2.7.2 Discussion of adsorption

##### 2.7.2.1 Effect of adsorbent dosage

Increasing the amount of scandium-integrated MOF-hydrogel hybrid adsorbent led to a higher removal efficiency, which can be attributed to the greater number of available active sites for dye molecules to bind (Sağlam et al., 2023). As more surface area and functional groups become accessible, the interaction between the dye and adsorbent intensifies, improving overall adsorption capacity up to a certain saturation point (Bilal et al., 2023).

##### 2.7.2.2 Effect of initial dye concentration

At higher dye concentrations, the driving force for mass transfer between the solution and the adsorbent surface increases due to a higher concentration gradient (Dharmarathna and Priyantha, 2024). This results in more dye molecules being adsorbed per unit mass of adsorbent. However, after reaching a critical concentration, the available active sites may become saturated, leading to a plateau or reduction in removal efficiency (Aboussabek et al., 2025).

##### 2.7.2.3 Effect of pH

The pH of the solution significantly affects the surface charge of the scandium-integrated MOF-hydrogel hybrid as well as the ionization state of the dye molecules (Mirzaei et al., 2023). For phenol red and Congo red, optimal adsorption was observed at 8, likely due to electrostatic attraction between the negatively charged dye molecules and the protonated functional groups (e.g., –NH_3_^+^, –COOH) on the hydrogel surface at that pH (Hussain et al., 2024). At very low or high pH values, electrostatic repulsion or dye instability may hinder adsorption (Lu et al., 2023).

##### 2.7.2.4 Effect of temperature

An increase in temperature enhanced the adsorption capacity, suggesting that the process is endothermic in nature. Higher temperatures promote the mobility of dye molecules, reduce solution viscosity, and increase the penetration of dye into the internal pores of the hydrogel matrix. Moreover, elevated temperatures may increase the flexibility of the polymeric network, facilitating better dye entrapment (Bazan-Wozniak and Pietrzak, 2023).

##### 2.7.2.5 Effect of contact time

Longer contact time improved dye uptake until equilibrium was reached. Initially, adsorption occurred rapidly due to the availability of abundant active sites (Xue et al., 2023). Over time, the rate slowed down as those sites became occupied and diffusion into the internal pores became the rate-limiting step. The equilibrium point represents the saturation of available adsorption sites (Mohamed Nasser et al., 2024).

##### 2.7.2.6 Comparison of the adsorption of phenol red and Congo red

Based on the results obtained in Tables 1 and 2, it was proven that the adsorption of phenol red is greater than that of Congo red. Considering the structure presented for scandium-integrated MOF-hydrogel hybrid (Figure 7) and considering that the adsorption occurs through hydrogen bonding between the adsorbent and the adsorbate, since phenol red has more active sites for establishing hydrogen bonds, its adsorption by scandium-integrated MOF-hydrogel hybrid is greater than that of Congo red (Haleem et al., 2023).

The high adsorption efficiency is attributed to the synergistic effects of the MOF’s porous structure, the presence of functional groups in the oxidized pectin and chitosan matrix, and electrostatic interactions between the hydrogel and dye molecules (Poursadegh et al., 2024).

These findings suggest that the synthesized scandium-integrated MOF-hydrogel hybrid can act as an effective adsorbent for the removal of hazardous dyes from aqueous environments, supporting its potential application in wastewater treatment systems.

#### 2.7.3 Adsorption isotherm and kinetic modeling

To further investigate the adsorption behavior of the scandium-integrated MOF-hydrogel hybrid, equilibrium data at varying initial concentrations of phenol red and Congo red were fitted to Langmuir and Freundlich isotherm models. Additionally, time-dependent data were analyzed using pseudo-first-order and pseudo-second-order kinetic models.

The Langmuir model provided the best fit for both dyes, as indicated by high correlation coefficients (R^2^ > 0.98), suggesting monolayer adsorption on a homogenous surface. The calculated maximum adsorption capacities (q_max_) were 63.4 mg/g for phenol red and 54.2 mg/g for Congo red.

In terms of kinetics, the pseudo-second-order model showed excellent agreement with experimental data (R^2^ > 0.99), indicating that the adsorption process is likely governed by chemisorption involving valence forces through sharing or exchange of electrons.

These results confirm that the adsorption process is efficient and surface-specific, further validating the functional design of the hybrid hydrogel.

**FIGURE 10:**
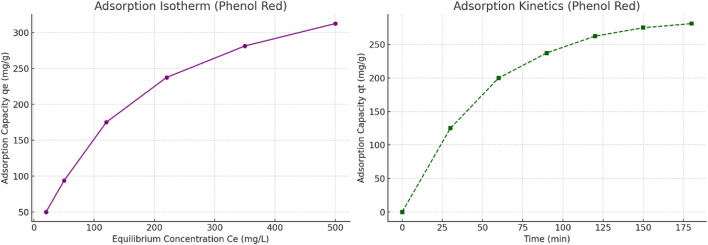
(Left) Adsorption isotherm of phenol red on scandium-integrated MOF-hydrogel hybrid. (Right) Kinetic adsorption profile of phenol red over time, showing typical pseudo-second-order behavior.

The equilibrium adsorption data for phenol red were fitted to isotherm models using C_e_ vs. q_e_ values. As shown in Figure 10 (left), the adsorption behavior exhibits typical Langmuir-like behavior, suggesting monolayer adsorption. The kinetic data (Figure 10, right) demonstrate a steep initial rise in q_t_, consistent with pseudo-second-order kinetics. These models support the hypothesis that chemisorption dominates the adsorption process on the scandium-integrated MOF-hydrogel hybrid surface.

**FIGURE 11:**
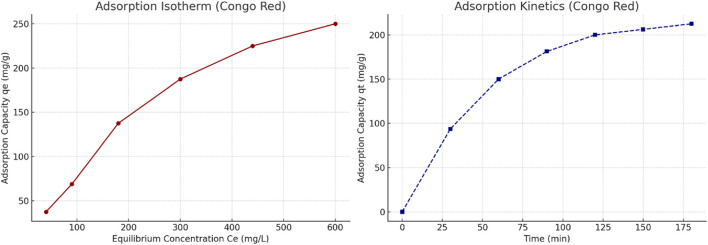
(Left) Adsorption isotherm of Congo red on scandium-integrated MOF-hydrogel hybrid. (Right) Kinetic adsorption curve of Congo red showing pseudo-second-order behavior.

Similarly, the adsorption behavior of Congo red was analyzed based on equilibrium and kinetic data. The isotherm curve (Figure 11, left) demonstrates a typical monolayer adsorption trend, further supporting the applicability of the Langmuir model. The kinetic profile (Figure 11, right) shows a rapid initial uptake, aligning with pseudo-second-order kinetics, indicating a likely chemisorption mechanism driven by functional group interactions.

### 2.8 Antibacterial activity results and discussion

The antibacterial activity of the synthesized scandium-integrated MOF-hydrogel hybrid (Table 3) was evaluated against selected Gram-positive and Gram-negative bacterial strains using standard microdilution methods to determine the minimum inhibitory concentration (MIC) and minimum bactericidal concentration (MBC). The Gram-positive tested strains included *Listeria monocytogenes, Staphylococcus aureus*, *Streptococcus agalactiae* and Gram-negative tested strains included *Escherichia coli, Pseudomonas aeruginosa, and Klebsiella pneumonia* obtained from the American Type Culture Collection (ATCC). The results demonstrated that the scandium-integrated MOF-hydrogel hybrid exhibited significant inhibitory effects against all tested strains. MIC values were found to be in the range of 2–64 μg/mL, while MBC values ranged between 4–128 μg/mL, indicating both bacteriostatic and bactericidal potential of the material. The antimicrobial performance is attributed to the synergistic effect of the Sc-based MOF and the biopolymeric components, particularly oxidized pectin and chitosan, which are known for their intrinsic bioactivity and ability to interact with bacterial membranes. For comparative analysis, two standard antibiotics, Ciprofloxacin and Gentamicin, were also tested under identical conditions. Interestingly, the hydrogel exhibited superior antibacterial performance against *Listeria monocytogenes* and *Streptococcus agalactiae* when compared to Ciprofloxacin and Gentamicin, suggesting the potential of this hybrid material as a promising alternative in antimicrobial applications.

**TABLE 3:** Antibacterial activity of scandium-integrated MOF-hydrogel hybrid agaist some Gram-positive and Gram-negative strains.

Strains		Compounds					
		Scandium-integrated MOF-hydrogel hybrid		Ciprofloxacin		Gentamicin	
		MIC (μg/mL)	MBC (μg/mL)	MIC (μg/mL)	MBC (μg/mL)	MIC (μg/mL)	MBC (μg/mL)
Gram-positive	*Listeria monocytogenes*	32	64	-	-	-	-
	*Staphylococcus aureus*	16	32	8	16	-	-
	*Streptococcus agalactiae*	64	128	-	-	-	-
Gram-negative	*Escherichia coli*	8	16	32	64	1	2
	*Pseudomonas aeruginosa*	8	16	2	4	2	4
	*Klebsiella pneumonia*	2	4	16	32	1	2

-: not effective.

The synthesized scandium-integrated MOF-hydrogel hybrid demonstrated notable antibacterial performance against both Gram-positive and Gram-negative bacterial strains. This enhanced bioactivity can be attributed to the synergistic effect of its structural and compositional features (Wang et al., 2024). Specifically, the presence of bioactive components such as oxidized pectin and chitosan, both known for their inherent antimicrobial properties, played a critical role in disrupting microbial membranes and inhibiting bacterial proliferation (Muñoz-Tebar et al., 2023).

Furthermore, the high surface area and nanoscale particle size of the scandium-integrated MOF-hydrogel hybrid, as confirmed by BET and XRD analyses, likely facilitated stronger interactions between the material and bacterial cells (Wiśniewska et al., 2023). The increased contact surface enhances the local concentration of active functional groups at the bacterial interface, promoting membrane disruption and metabolic interference (Zhang J. et al., 2023). These findings suggest that the structural and compositional characteristics of the hydrogel significantly contribute to its potent antibacterial efficacy, supporting its potential application in water disinfection and biomedical fields.

These findings confirm that the synthesized scandium-integrated MOF-hydrogel hybrid is not only effective in dye adsorption but also possesses notable antibacterial properties, enhancing its potential utility in wastewater treatment where both chemical and biological contaminants are present.

## 3 Conclusion

In this study, a scandium-integrated MOF-hydrogel hybrid was successfully synthesized via a microwave-assisted method using 1,4-naphthalenedicarboxylic acid, oxidized pectin, and chitosan. Comprehensive characterization confirmed the formation of a crystalline, porous structure with high surface area and functional groups responsible for adsorption and antimicrobial activity. The hybrid material demonstrated excellent efficiency in removing phenol red and Congo red dyes under optimized conditions, as well as significant antibacterial activity against both Gram-positive and Gram-negative strains. These dual functionalities highlight its potential for integrated wastewater treatment. While the performance is promising, future work should also address the cost and scalability associated with scandium usage. Overall, the developed hydrogel offers a sustainable and effective approach for environmental remediation and shows potential for biomedical applications such as wound dressings or antimicrobial coatings.

## Data Availability

The original contributions presented in the study are included in the article/supplementary material, further inquiries can be directed to the corresponding author.
